# Surveillance of physical activity and sedentary behaviour in czech children and adolescents: a scoping review of the literature from the past two decades

**DOI:** 10.1186/s12889-022-12766-0

**Published:** 2022-02-21

**Authors:** Eliška Materová, Jana Pelclová, Aleš Gába, Karel Frömel

**Affiliations:** 1 Institute of Active Lifestyle, Faculty of Physical Culture, Palacký University Olomouc, tř. Míru 117, 771 11 Olomouc, Czech Republic; 2 Department of Natural Sciences in Kinathropology, Faculty of Physical Culture, Palacký University Olomouc, tř. Míru 117, 771 11 Olomouc, Czech Republic; 3 Institute of Sport Sciences, the Jerzy Kukuczka Academy of Physical Education, Katowice, Poland

**Keywords:** Prevalence, Insufficient physical activity, Youth, Health

## Abstract

**Background:**

This study aimed to map the available evidence related to physical activity (PA) and sedentary behaviour (SB) in Czech children and adolescents and suggest future directions and improvements to strengthen the surveillance of PA and SB in the Czech Republic.

**Methods:**

The search of articles published between January 2000 and December 2020 included the Medline and Medvik databases and a manual search in eight Czech journals related to the topic. This review followed the “Preferred Reporting Items for Systematic reviews and Meta-Analyses extension for Scoping Reviews”.

**Results:**

Out of 350 identified articles, 79 articles met the criteria for selection and referred to 27 studies. The majority of the articles were cross-sectional (89%), approximately two-thirds of the articles (61%) examined only PA, and half of the articles (51%) employed device-based assessments. Approximately 47% of the articles reported the prevalence of physical inactivity on the basis of inconsistently defined recommendations. Approximately 14%, 23%, and 10% of the articles focused on active transportation, organized PA (including physical education or leisure-time PA), and parent-child PA, respectively.

**Conclusions:**

Future studies need to focus on longitudinal design and interventions, randomly selected samples, a mix of device-based and self-reported methods, and the recognition of health-related 24-hour time use patterns. This review advocates the government-supported development of a national surveillance system that will help to reduce insufficient PA and excessive SB.

**Supplementary Information:**

The online version contains supplementary material available at 10.1186/s12889-022-12766-0.

## Background

In children and adolescents, physical activity (PA) is an important part of a healthy lifestyle. Sufficient PA is associated with improved physical fitness, cardiometabolic health, bone health, cognitive outcomes, and mental health, and reduces adiposity [1, 2]. Conversely, insufficient PA accompanied by high levels of sedentary behaviour (SB) has a negative influence on health [3] and together they are considered as the key drivers of non-communicable diseases [4] with a possible transfer to adulthood [5]. Currently, the Global Action Plan on PA 2018–2030, the mission of which is to ensure access to safe and enabling environments and to diverse opportunities to be physically active for all people, draws attention to the fact that 81% of adolescents are not sufficiently active [6]. Moreover, the prevalence of insufficient engagement in everyday PA increases during adolescence and seems to be higher among girls [7].

PA is complex movement behaviour and might be performed with varying intensities, body postures, domains, and bout duration, and for multiple reasons. In many countries across the globe, the relevant issues related to insufficient PA have resulted in the development of national PA guidelines which provide a consensus on the amount, intensity, frequency, and type of PA necessary for the prevention of chronic disease and for supporting collective health [8]. Besides promoting an optimal level of PA, limiting SB is also recommended in the majority of these countries. SB is defined as any waking behaviour characterized by an energy expenditure ≤1.5 metabolic equivalents (METs) while in a sitting, reclining, or lying posture [9]. Specifically, time spent using a device such as a computer, television, or games console, which is called “screen time” (ST), is a more specific component of total SB with several negative effects on health [10]. Achieving the required level of PA and SB offers significant health benefits and mitigates health risks which have been recently highlighted by the World Health Organization (WHO) in the most recent edition of the “WHO Guidelines on Physical Activity and Sedentary Behaviour” [1].

The WHO also announced national surveillance of PA as an essential part of the global initiatives [1]. A comprehensive surveillance system is required to monitor PA levels accurately at the population level. It provides essential information on compliance with the PA and SB guidelines and allows determinants and trends over time to be identified [11]. This information is required to guide, monitor, and evaluate policies and intervention programmes aimed at improving the level of PA among children and adolescents [12]. Moreover, the national data could help to adopt global PA and SB recommendations with appropriate adjustment to the social, cultural, and environmental conditions of the state.

In the Czech Republic, a national surveillance system, as well as country-specific recommendations for children’s and adolescentsʼ PA, is lacking. The absence of PA surveillance is partly compensated for by the national “Report Card on Physical Activity for Children and Youth”, the first edition of which was published in 2018 [13, 14]. Although the report card presents estimates of the level of physical inactivity and excessive ST that are based on the synthesis of the available evidence from 2013 to 2017, it draws attention to the inadequacy that exists in the area of PA research.

The inadequacy in the area of PA research is also visible in a recently published systematic review of global, regional, and national trends and patterns in PA research since 1950, in which the Czech Republic, with a total of 105 papers, is in nineteenth place among high-income European countries [15]. Likewise, no study has summarized and reviewed existing research on PA and SB in Czech children and adolescents. Such a comprehensive analysis describing up-to-date surveillance and its main findings is critical as a prelude to further steps which could lead to the development of a national surveillance system and recommendations.

Therefore, the purpose of this study was to systematically review the available evidence on PA and SB in Czech children and adolescents published in the last two decades. Specifically, we aimed to (1) summarize the descriptive characteristics and main conclusions of the available evidence and (2) suggest future directions and improvements to strengthen the surveillance of PA and SB in Czech children and adolescents.

## Methods

### The search strategy

This scoping review conforms to the “Preferred Reporting Items for Systematic reviews and Meta-Analyses extension for Scoping Reviews” (PRISMA-ScR) [16]. The checklist was followed in reporting this study (Supplementary file 1). The search included the Medline (via Ovid) and Medvik databases. We searched for studies published from January 2000 to December 2020 but only studies whose data was obtained during this period were included in the review. The search strategy was arranged in three sets of terms that cover the target population (i.e. school-aged children), movement behaviours (i.e. PA and SB), and country (the Czech Republic). The search strategy builds mainly on the Medical Subject Heading (MeSH) terms. Subsequently, selected free-text terms were included in the search strategy to increase the sensitivity of the search. The complete search strategy used in the electronic databases is presented in Supplementary file 2. The search results were imported into the EndNote program (Version X7; Thomson Reuters, San Francisco, CA, USA), all duplicates were deleted, and an online Excel spreadsheet was produced for screening purposes. The final database included several unique identifiers for each study and several items of bibliographical information (article identifier, authors, year of publication, study title, abstract, and journal title).

Next, the title and abstract of the studies were screened by two independent evaluators. The studies were divided into three groups: relevant, irrelevant, and potentially relevant studies. In the case of potentially relevant studies, their full texts were analysed, and then the studies were included or excluded. In the event of a disagreement between the evaluators concerning the relevance of a particular study, the final decision as to inclusion or exclusion was made by a third person.

In addition to searching the databases mentioned above, the authors performed a manual search in the references of studies included in the review. A manual search was also carried out in the main Czech journals related to the topic of interest and which were not indexed in the Medline and Medvik databases during part or the whole of the past 20 years. Specifically, we performed a manual search in Tělesná kultura, Acta Gymnica, Česká Kinantropologie, Studia Kinanthropologica, Medicina Sportiva Bohemica et Slovaca, Tělesná výchova a sport mládeže, Acta Universitatis Carolinae – Kinanthropologica, and Studia Sportiva.

### Inclusion and exclusion criteria for studies

 Articles with any research design were included in this scoping review if they: (a) targeted Czech children and adolescents aged from six to 20 years, (b) reported PA (overall PA, organized sports and PA, leisure-time PA, physical fitness, steps per day, active transportation, cycling, walking, roller-skating, training, physical education lessons, active play, locomotion, or movement), SB (total sedentary time, ST, physical inactivity, sitting, or lying), or both, (c) were written in the English or Czech language, (d) were published between 2000 and 2020, and (e) were published as a peer-reviewed journal article. International studies without a clearly defined Czech sample were excluded. The minimum sample size was set to at least 50 participants for cross-sectional studies but none was set for longitudinal studies.

### Data extraction

The basic characteristics about the studies that were included (design, primary outcomes, and main findings) and descriptive characteristics of the study samples (sample size, percentage of girls, age category or average age, and region of the Czech Republic) were extracted. We also extracted information on the use of methods used for measurement (self-reported, device-measured) and dealt with more detailed information on PA and SB. Finally, we summarized the main findings and results of the studies that were included.

## Results

### Data extraction procedure and bibliographic characteristics

**Fig. 1:**
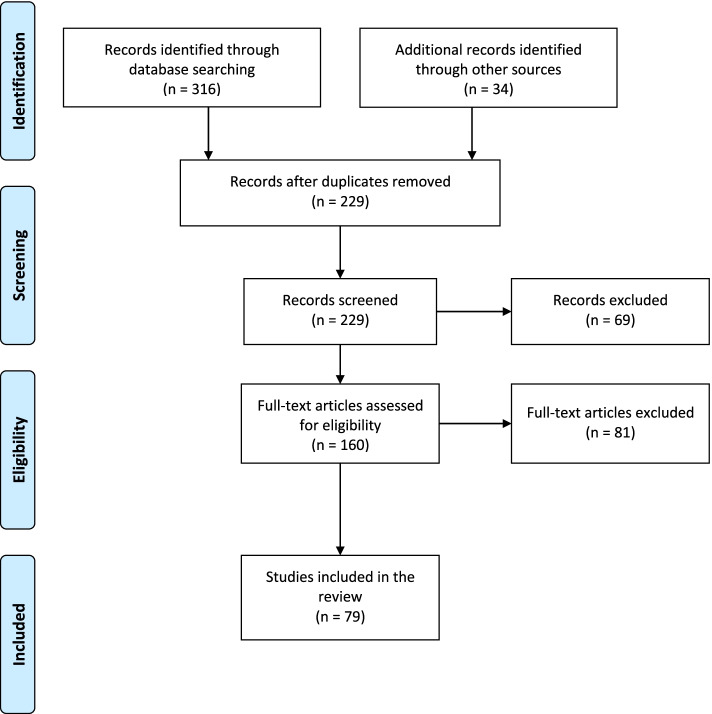
The flow of information through the different phases of the review.

In the Medline and Medvik databases, a total of 316 articles were identified on the basis of the search strategy, and 34 articles were identified by the manual search (Fig. 1). After the removal of duplicates, the exclusion of some articles on the basis of predetermined criteria was performed. Out of 160 full-text articles assessed for eligibility, there was a disagreement between the evaluators concerning the relevance of a particular study in eight cases, and the final decision as to whether to include or exclude them was made by a third person. Although these papers were related to the PA and SB of Czech children and adolescents, the main reasons for exclusion were: (a) the impossibility of separating the Czech results from the results of other countries [17], (b) PA and SB results related to very specific samples (with a different mental load or level of academic achievement) without clear summary findings [18–20], and (c) specific studies examined test-retest reliability [21, 22].

The final sample included 79 articles [23–77, 79–102] referring to 27 studies. An overview of all articles is shown in Table 1. Out of 79 articles, 27 referred to the WHO study called Health Behaviour in School-aged Children (HBSC) with the data collections in 2009/2010 [29, 59, 61, 80, 83, 87, 101], 2013/2014 [23, 32, 35, 57, 79, 89, 91–93, 96, 98], 2017/2018 [31, 88] or in different periods within the trend analyses [30, 36, 58, 63, 64, 84, 86]. Six articles belonged to the intervention study in school-aged children in Olomouc region [42, 43, 49–51, 100], 2 articles were part of the Moravian study [52, 53], 3 articles are part of the 24-h movement behaviour study [58, 62, 81], 3 articles referred to one-year longitudinal study [39, 47, 48]. Nine articles were published within parents-children study [33, 72, 73, 76, 77, 82, 85, 95, 102]. Two articles were part of adolescent survey [67, 88], 2 articles referred to ActiTrainer accelerometer-based children study [65, 94], 3 articles referred to ActiTrainer accelerometer-based adolescent study [27, 28, 69], and 2 articles were related to 15-16year adolescent study [68, 90]. Six articles referred to adolescent study using pedometers and International Physical Activity Questionnaire (IPAQ) [24–26, 46, 60, 75]. Sixteen articles presented findings from independent studies [34, 37, 38, 40, 41, 44, 45, 54–56, 66, 70, 71, 74, 97, 99].

**Table 1:** Characteristics and findings of the studies included

First author and year of publication	Characteristics of study sample	Methods	Primary outcome(s)	Main finding(s)
**Baďura et al., 2018** [89]	n = 6935^d^ 51% girls 13–15 years Czech Republic	Self-reported (HBSC questionnaire)	PA (OLTA (team sports, individual sports and other activities))	79% of the respondents engaged in at least one OLTA per week. 34% of the respondents engaged in overall unstructured activities daily or at least twice a week. The selected unstructured activities were strongly associated with an increased occurrence of adolescents’ health-risk behaviours and low academic achievement.
**Baďura et al., 2017** [91]	n = 10,279^d^ 51% girls 11–15 years Czech Republic	Self-reported (HBSC questionnaire)	PA (OLTA (team sports, individual sports and other activities))	48% of the boys and 52% of the girls participated in OLTA. With increasing age, participation in OLTA decreases. OLTA participation was associated with a lower occurrence of repeated substance use and truancy and inversely with higher odds of physical fights and injuries. Girls, in general, were at lower risk when participating in OLTA than boys.
**Baďura et al., 2016** [92]	n = 10,483^d^ 51% girls 11–15 years Czech Republic	Self-reported (HBSC questionnaire)	PA (OLTA (team sports, individual sports and other activities))	Involvement of adolescent in OLTA was linked to general better school performance and attachment to school. Adolescents participating in more activities at the same time had the best school performance.
**Baďura et al., 2015** [93]	n = 10,503^d^ 51% girls 11–15 years Czech Republic	Self-reported (HBSC questionnaire)	PA (OLTA (team sports, individual sports and other activities))	81% of the respondents participated in one or more OLTA. Participation in OLTA was associated with better physical and mental health in adolescents. The association varies according to the pattern of participation in activity and was partly gender- and age-specific.
**Bešič et al., 2016** [44]	n = 174 48% girls 9–11 years Olomouc region	Device-measured (ActiGraph GT3X and GT3X+ accelerometers, hip-worn, 7 consecutive days, >10 valid hours per day, at least 4 weekdays and 1 weekend day, CoP by Freedson, 2005)	PA (total, LPA, MPA, VPA)	The boys had higher BMI, spent more time on VPA, and showed better results in health-related physical fitness than the girls. Those children who spent more time on VPA were more likely to have better cardiorespiratory fitness.
**Bláha et al., 2019** [54]	n = 639 46% girls 11–16 years North-western Bohemia region	Device-measured (Digi-walker Yamax SW-200 and SW-700 pedometers, at least 5–6 weekdays and 1 weekend day)	PA (steps per day)	The boys achieved more steps per day (by 1000 steps) than the girls. Weekday steps per day were significantly higher than weekend steps per day.
**Bláha et al., 2010** [55]	n = 1185 59% girls 11–16 years North-western Bohemia region	Self-reported (PA and SB questionnaire)	PA (OLTA, PI)	The participation in OLTA was, on average, 8% higher in the boys than the girls on all days of the week and decreased with age, particularly in the girls. The boys had 6% higher ST than the girls on all days of the week.
**Bucksch et al., 2019** [23]	n = 5082^d^ 52% girls 11–15 years Czech Republic^b^	Self-reported (HBSC questionnaire)	PA (MVPA) SB (ST)	Meeting the MVPA recommendation was reported by 21.5% of Czech adolescents. Low (less than 3.5 h per school day), medium (3.5-7), and high (more than 7) ST levels were reported by 30.7%, 35%, and 34.4% of Czech adolescents, respectively. The social and environmental correlates differed for the MVPA and ST of the adolescents.
**de Gouw et al., 2010** [41]	n = 30,966^d^ 56% girls 10–18 years Czech Republic	Self-reported (questionnaire)	PA (total, up to 7 h per week or > 7 h per week) SB (ST)	60% of the respondents performed PA during the week. The boys were more physically active, watched more television, and used a computer more often than the girls. Watching TV for more than 7 h a week was positively associated with being overweight/obese in 15-18-year-old girls and was found to be negatively associated in boys of the same age group.
**Dygrýn et al., 2015** [34]	n = 6236 50% girls 12–17 years Olomouc and Hradec Králové regions	Self-reported (national 2011 Czech Census of Population and Housing)	PA (AT – commuting to/from school)	A decrease in the proportion of active commuting to school between 2001 and 2011 was observed in 47% of the respondents. Between 2001 and 2011, the proportion of adolescents actively commuting to school decreased by 47%, from an absolute rate of 49.1–26%. The proportion of active commuters fell in low-walkable areas by 61% and in high-walkable areas by 39%. The adolescents were 2.7 times less likely to commute actively in 2011 than in 2001.
**Frömel et al., 2020a** [24]	n = 1462 64% girls 15–19 years Czech Republic^b^	Self-reported (IPAQ-LF)	PA (total, VPA, MVPA, AT)	A higher rate of AT was only significantly associated with higher well-being in the girls. AT accounted for 22.5% and 24.9% of the weekly PA of Czech boys and girls, respectively.
**Frömel et al., 2020b** [75]	n = 596 62% girls 15–19 years Czech Republic	Device-measured (Digi-walker Yamax SW-700 pedometer) Self-reported (IPAQ-LF)	PA (steps per day)	The stronger negative associations between depressive symptoms and PA (especially recreational PA) were confirmed. The girls who reported the fewest depressive symptoms had 2.12 times greater odds of meeting the 11,000 steps per day recommendation than did the girls with the most depressive symptoms.
**Frömel et al., 2018** [25]	n = 1117 60% girls 15–17 years Czech Republic^b^	Self-reported (IPAQ-LF)	PA (total, VPA, MPA, LPA, MVPA)	The girls were found to be less active than the boys at high PA intensities. The recommendations for vigorous PA were met by 46% of the Czech boys and 33% of the girls.
**Frömel et al., 2017** [26]	n = 6371 60% girls 15–17 years Czech Republic^b^	Device-measured (Digi-walker Yamax SW-700 pedometer, at least 3 weekdays and 1 weekend day) Self-reported (IPAQ-LF, PA preferences questionnaire)	PA (total, VPA, MVPA, PA preferences, steps per day)	Agreement between preferred PA and that actually undertaken was associated with higher odds of meeting the weekly PA recommendations and higher levels of well-being both in boys and girls.
**Frömel et al., 2016a** [69]	n = 641 68% girls Mean age: 16.6 years Czech Republic	Device-measured (ActiTrainer accelerometer, hip-worn, 3 school days; Digi-walker Yamax SW-700 pedometer; Polar heart rate sensor)	PA (school, MVPA during PE lessons and recess, steps per hour, steps per day)	Both the boys and girls participating in PE lessons reported significantly better results compared with non-participating individuals regarding all indicators of volume and intensity of school PA. An increase in school PA and an improved lifestyle in the adolescents on school days were supported significantly more by PE lessons than by a longer recess time.
**Frömel et al., 2016b** [27]	n = 236 70% girls Mean age: 16.0 years Czech Republic^b^	Device-measured (ActiTrainer accelerometer, hip-worn, at least 1 school day and 1 weekend day, 15-s epoch; Polar heart rate sensor)	PA (total and school steps per hour, PI)	Both the boys and girls participated in more PA at lower intensities at weekends compared with school days. The time spent sitting (lying) in front of a computer was 60 min per day longer in the boys than in the girls.
**Frömel et al., 2007** [71]	n = 440 53% girls 15–20 years Czech Republic	Self-reported (IPAQ-LF)	PA (total and school, VPA, OLTA)	The boys were more active than the girls. Increasing age was associated with lower OLTA, AT, and school or work PA in the boys, and lower leisure-time and higher PA in the household and at school or work in the girls.
**Gába et al., 2020a** [81]	n = 659 58% girls 8–18 years Czech Republic	Device-measured (ActiGraph GT9X Link or wGT3X-BT accelerometer, non-dominant wrist, 7 consecutive days, >16 valid hours per day, at least 4 weekdays and 1 weekend day)	PA (multi-day 24-h data; LPA, MVPA) SB (total sedentary time)	The children had more LPA than the adolescents by 26 min per day and more MVPA by 19 min per day. The adolescents had more SB than the children by 111 min per day. In the children, being a short sleeper was associated with higher SB by 95 min per day and lower MVPA by 16 min per day. In the adolescents, being a short sleeper was associated with a higher amount of time spent in SB by 67 min per day and lower LPA by 2 min per day.
**Gába et al., 2020b** [52]	n = 425 58% girls 7–12 years Moravia region	Device-measured (ActiGraph GT3X accelerometer, hip-worn, 7 consecutive days, 60-s epoch, >10 valid hours per day, at least 4 weekdays and 1 weekend day, CoP by Evenson)	PA (total, LPA, MVPA) SB (total sedentary time)	The children spent 87% of their total SB time in sedentary bouts that were shorter than 30 min. The boys spent on average 9.9 min per day less in short sedentary bouts and 7.5 min per day more in long sedentary bouts compared with the girls. Adiposity status could be improved by increasing MVPA at the expense of time spent in medium-length sedentary bouts. Some benefits for adiposity were found for replacing middle sedentary bouts with short sedentary bouts.
**Gába et al., 2017** [53]	n = 365^d^ 57% girls 7–12 years Moravia region	Device-measured (ActiGraph GT3X accelerometer, hip-worn, 7 consecutive days, 60-s epoch, >10 valid hours per day, at least 3 weekdays and 1 weekend day, CoP by Evenson)	PA (total, LPA, MVPA, VPA, steps per day) SB (total sedentary time)	In terms of their overall PA, the boys were more active than the girls. No associations were found between LPA and all body fatness indicators. MVPA was negatively associated with all body fatness indicators only in the girls. In contrast, vigorous PA was strongly negatively associated with body fatness indicators only in the boys.
**Groffik et al., 2020** [28]	n = 629 66% girls Mean age: 16.2 years Czech Republic^b^	Device-measured (ActiTrainer accelerometer, hip-worn, 4 consecutive days, at least 1–3 days, 15-s epoch; Digi-walker Yamax SW-700 pedometer; Polar heart rate sensor) Self-reported (IPAQ-LF)	PA (total and school, MVPA, steps/hour)	Participation in PE lessons was associated with a higher rate of meeting school PA recommendations. Compared with the Czech Republic, more PE lessons in the Polish education system were associated with increased daily VPA and a greater portion of school PA in daily PA.
**Hamřík et al., 2015** [29]	n =4404^d^ 52% girls 11–15 years Czech Republic^b^	Self-reported (HBSC questionnaire)	PA (total, VPA) SB (ST)	VPA was positively associated with the healthy development of adolescents. Screen-based behaviour showed an inverse relationship with adolescents’ healthy development, especially in the group of 11- and 13-year-old children.
**Hamřík et al., 2014** [80]	n = 4365^d^ 52% girls 11–15 years Czech Republic	Self-reported (HBSC questionnaire)	SB (ST)	The prevalence of SB rises with growing age, with the most visible increase in prevalence between age 11 and age 13. SB was significantly more prevalent among the adolescent boys compared with the adolescent girls. Czech adolescents were more likely not to meet the recommendation of 2 h for watching TV at weekends compared to weekdays. Playing computer games was more common among the boys, contrary to chatting online, which was more common among the girls.
**Hamřík et al., 2012** [83]	n = 4425^d^ 52% girls 11–15 years Czech Republic	Self-reported (HBSC questionnaire)	SB (ST)	More than 55% of the girls and 60% of the boys spent more than 2 h a day in front of a TV, DVD, or video screen in the working week. With age, the proportion of children who spent 2 or more hours a day using a computer increases.
**Hollein et al., 2018** [101]	n = 4404 52% girls 11–15 years Czech Republic	Self-reported (HBSC questionnaire)	PA (AT – commuting to/from school)	58% of the children used AT for commuting to and from school. Children actively commuted more often in schools which had formally processed health promotion objectives than in schools without these goals. In addition, children who had a school in the same village or city had a higher chance of AT than children who had a school elsewhere than in their place of residence.
**Hollein et al., 2017** [87]	n = 1522 52% girls 15 years Czech Republic	Self-reported (HBSC questionnaire)	PA (AT – commuting to/from school)	School policies and programmes promoting AT to and from schools in the Czech Republic contributed to the use of AT. The association between school policies and programmes and AT was stronger in the boys compared to the girls.
**Jakubec et al., 2020** [58]	n = 679 57% girls 8–18 years Czech Republic	Device-measured (ActiGraph GT9X Link or wGT3X-BT accelerometer, non-dominant wrist, 7 consecutive days, >16 valid hours per day, at least 3 weekdays and 1 weekend day) Self-reported (HBSC questionnaire)	PA (multi-day 24-h data; LPA, MVPA) SB (ST)	No associations were found between meeting all three recommendations (≥60 min per day of MVPA, < 2 h per day of recreational ST, and uninterrupted sleep for 9–11 h per day (for children) or 8–10 h per day (for adolescents) within the 24-hour movement guidelines and adiposity indicators. However, meeting only the ST recommendation and the combination of the ST and sleep recommendations was associated with a reduced risk of excess adiposity.
**Kalman et al., 2015a** [59]	n = 4385^d^ 52% girls 11–15 years Czech Republic	Self-reported (HBSC questionnaire)	PA (total, MVPA, VPA, motives for PA)	A substantial part of the boys and girls were not participating in MVPA and VPA as recommended. MVPA and VPA among the girls significantly decreased from age 11 to age 15. Compared to the girls, the boys reported significantly more MVPA and VPA in all age groups, except 11-year-old adolescents, where the levels of MVPA did not differ between the girls and boys. The girls appear to be more influenced by social motives. The importance of these motives became higher with increasing age. Achievement motivation for PA was more important for the boys and it also increased with age.
**Kalman et al., 2015b** [30]	n = n/a^d,e^ 11–15 years Czech Republic^b^	Self-reported (HBSC questionnaire)	PA (total, MVPA)	In Czech adolescents, there was a decrease in meeting the MVPA recommendation of at least 60 min daily (4% and 3% for boys and girls, respectively) between 2002 and 2010.
**Kleszczewska et al., 2020** [31]	n = 11,553 11–15 years Czech Republic^b^	Self-reported (HBSC questionnaire)	PA (AT – commuting to/from school)	In the Czech sample, passive means of transport, walking, and cycling were found in 35, 62.1, and 2.9% of the adolescents, respectively. Cycling to school was protective against reports of health complaints. Adolescents who commuted to school actively were less likely to report especially psychological symptoms.
**Kokko et al., 2018** [32]	n = 10,501^d^ 11–15 years Czech Republic^b^	Self-reported (HBSC questionnaire)	PA (total, VPA, OLTA)	62% of the Czech children and adolescents (boys: 70%; girls: 55%; 11-, 13-, and 15-year-olds: 66, 64, and 57%, respectively) took part in OLTA. Participants in OLTA were more likely to meet the overall PA recommendations and VPA recommendations than non-participants.
**Kopčáková et al., 2017** [35]	n = 3481^d^ 11–15 years Czech Republic^b^	Self-reported (HBSC questionnaire)	PA (total, MVPA) SB (ST)	An environment perceived as activity-friendly was associated with higher odds of adolescents meeting the recommendations for PA and lower odds of excessive screen-based activities.
**Kudláček et al., 2020** [46]	n = 2334 65% girls 15–18 years Czech Republic^b^	Device-measured (Digi-walker Yamax SW-700 pedometer) Self-reported (IPAQ-LF, QPAP)	PA (steps per day, VPA, MVPA, preferences for fitness PA)	The preference for fitness PA in the boys was highly stable over the 8-year study period, with fitness PA ranked third after team and individual PA. For the girls, an increasing trend was observed in the preference for fitness PA at the expense of dance and outdoor PA. In both the boys and girls, those who preferred fitness PA were more likely to achieve the recommended weekly PA level than those who did not prefer fitness PA.
**Kudláček, 2015** [45]	n = 238 55% girls Mean age: 17.0 years Olomouc and Hradec Králové regions	Self-reported (IPAQ-LF; questionnaire – Sport Preferences Survey)	PA (total, MPA, VPA, walking, leisure-time PA, school PA, PA preferences)	The students from Vrchlabí showed a significantly higher level of leisure-time PA than the students from Olomouc, who reported a significantly higher level of school-based PA.
**Maliňáková et al., 2018** [98]	n = 4182^d^ 51% girls Mean age: 14.4 years Czech Republic	Self-reported (HBSC questionnaire)	PA SB (ST)	Both spirituality and religious attendance of adolescents reduced the likelihood of excessive watching TV and playing computer games. Adolescent religious attendance and spirituality were associated with a more active way of spending leisure time (sporting and non-sporting activities and regularly reading books or playing a musical instrument).
**Miklánková et al., 2013** [99]	n = 124 6–11 years Czech Republic	Device-measured (Caltrac accelerometer, Digi-walker Yamax SW-200 pedometer, hip-worn, 7 consecutive days	PA (steps per day, active energy expenditure)	High levels of PA were found in all segments of the day and week (except weekend days) in the 10-11-year-old children compared to the 6-9-year-old children.
**Mitáš et al., 2020** [60]	n = 1908 59% girls 15–19 years Czech Republic	Device-measured (Digi-walker Yamax SW-700 pedometer; Polar heart rate sensor) Self-reported (IPAQ-LF)	PA (steps per day, MVPA)	Considering average steps per day and an achievement of 11,000 steps per day, a continuous significant decrease was found between 2010 and 2017 in the adolescent girls (by 18%) and boys (by 28%). The estimates of meeting the recommended weekly PA expressed as MET-min per week were not so convincing with regard to the decrease. Given the lowest amount of PA on Sunday, the combination of weekend days with Monday represents a great risk for young people in terms of health.
**Mitáš et al., 2009** [70]	n = 302 54% girls 14–15 years Czech Republic	Self-reported (IPAQ-LF)	PA (total, MPA, VPA, walking) SB (total sedentary time)	The girls were significantly more likely to be sitting than the boys. Children living in a middle-sized to large-sized community and living in an apartment were significantly more likely to be sitting.
**Ng et al., 2020** [57]	n = 4809^d^ 52% girls 11–15 years Czech Republic^b^	Self-reported (HBSC questionnaire)	PA (MVPA)	The Czech girls had better perceived school performance than the boys, and yet more boys than girls participated in daily MVPA. The associations between perceived school performance and MVPA showed an inverted U shape. The strongest association for very good perceived school performance was among young adolescents who reported 5 to 6 days of MVPA after controlling for family affluence scale.
**Nováková Lokvencová et al., 2011** [68]	n = 383 53% girls 15–16 years Czech Republic^b^	Device-measured (Digi-walker Yamax SW-700 pedometer, 7 consecutive days)	PA (total, steps per day)	The Czech boys and girls showed a significantly higher number of steps on school days than on weekend days. The differences in the daily number of steps between the Czech boys and girls were not significant on any day. The respondents reached the largest number of steps on Friday and the lowest number of steps on Sunday.
**Pavelka et al., 2017** [86]	n = 12,273 51% girls 11–15 years Czech Republic	Self-reported (HBSC questionnaire)	PA (AT – commuting to/from school)	AT to school decreased sharply among Czech children of school age from 2006 to 2014 (by 21.7% in the boys and by 23% in the girls). Walking was the most frequently used mode of AT. The boys were significantly more likely to cycle to school compared to the girls.
**Pavelka et al., 2016** [79]	n = 418^d^ 54% girls 11–15 years Olomouc region^b^	Self-reported (HBSC questionnaire)	SB (ST)	Two-thirds of the respondents watched television or used a computer for at least two hours a day. The older children spent excessive amounts of time watching television.
**Pavelka et al., 2012** [61]	n = 4425^d^ 52% girls 11–15 years Czech Republic	Self-reported (HBSC questionnaire)	PA (AT – commuting to/from school)	AT to and from school was opted for in the Czech Republic by approximately two-thirds of the children aged 11 to 15. Differences between genders were not significant; the largest percentage of children who opted for AT were aged 11 (69%). An important factor increasing the probability of AT by as much as 16 times was whether a child’s place of residence was in the same municipality as the school.
**Pelclová et al., 2010a**^**a**^ [47]	n = 13 85% girls Mean age: 15.6 years Olomouc region	Device-measured (Omron HJ-105 pedometer, waist, 10 months, >10 valid hours per day)	PA (steps per day, OLTA)	Regardless of the day, month, and season, the high school pupils who participated in regular OLTA achieved a mean of approximately 4000 more steps per day than the pupils who did not participate in after-school PA.
**Pelclová et al., 2010b**^**a**^ [48]	n = 12 83% girls Mean age: 16.0 years Olomouc region	Device-measured (Omron HJ-105 pedometer, hip-worn, 10 months, >10 valid hours per day)	PA (steps per day, school PA)	Across all months and seasons, high school pupils achieved notably more steps on weekdays than at weekends, and on PE days than on non-PE days. The total contribution of PE lessons (lasting 90 min) to the pupils’ daily PA was 10.0% additional steps per PE day. The lowest mean step counts were in February and the highest in June.
**Roubalová et al., 2018**^**a**^ [37]	n = 22 100% girls 6–11 years Moravia region	Device-measured (ActiGraph GT3X+ accelerometer, hip-worn, 7 consecutive days, 60-s epoch, >10 valid hours per day, at least 3 weekdays and 1 weekend day, CoP by Evenson)	PA (total, LPA, MVPA) SB (total sedentary time)	Seasonal differences were found in the volume of PA (LPA, MVPA) and SB in younger school-age girls. The highest values of SB were found in autumn (November). The lowest values of SB and the highest values of LPA and MVPA were found in spring (May). The younger girls reported lower values of SB and higher values of PA in all the seasons that were monitored than the older girls.
**Rubín et al., 2020** [62]	n = 679 56% girls 8–18 years Czech Republic	Device-measured (ActiGraph GT9X Link or wGT3X-BT accelerometer, non-dominant wrist, 7 consecutive days, >16 valid hours per day, at least 4 weekdays and 1 weekend day)	PA (multi-day 24-h data; LPA, MVPA) SB (ST)	Approximately 6.5% of the children and 2.2% of the adolescents met all the recommendations of the combined 24-h movement guidelines and several correlates related to family were identified. In the children, girls and participants with overweight or obese fathers had significantly lower odds of adherence to the combined movement guidelines.
**Sigmund et al., 2020a** [76]	n = 796 4–16 years Mean age: 10.0 years Czech Republic	Device-measured (Digi-walker Yamax SW-200 pedometer, 8 consecutive days, >6 valid hours per day)	PA (steps per day, OLTA) SB (ST)	The mother’s overweight/obesity significantly increases her children’s odds of overweight/obesity. Concerning fathers, active participation in OLTA and reaching 10,000 steps per day significantly reduce the odds of overweight/obesity in their children and adolescent offspring.
**Sigmund et al., 2020b** [82]	n = 1114^d^ 51% girls 6–16 years Czech Republic	Device-measured (Digi-walker Yamax SW-200 pedometer, hip-worn, 8 consecutive days, >8 valid hours per day), at least 4 weekdays and 2 weekend days)	PA (steps per day, OLTA)	Regardless of their parents’ overweight/obesity, the children who participated in OLTA ≥three times a week had a lower prevalence of obesity than the children without participation in OLTA (5.0% vs. 11.1%).
**Sigmund et al., 2018a** [77]	n = 649^d^ 51% girls Mean age: 9.3 years Czech Republic	Device-measured (Digi-walker Yamax SW-200 pedometer, hip-worn, 8 consecutive days, >8 valid hours per day), at least 4 weekdays and 2 weekend days)	PA (total, steps per day, OLTA) SB (ST)	The mother’s PA (achievement of at least 10,000 steps per day) was associated with the achievement of recommended daily steps in overweight/obese preschool and school-aged children.
**Sigmund et al., 2018b** [63]	n = 18,250^d^ 51% girls 10.5–16.5 years Czech Republic	Self-reported (HBSC questionnaire)	PA (MVPA) SB (ST)	A significant decrease was revealed in the rates of meeting the MVPA recommendation in the low-family affluence boys (from 28.9% in 2002 to 23.3% in 2014) and girls (22.3% in 2002 to 17.3% in 2014). A significant trend-related increase in excessive ST was evident in the adolescents, regardless of gender and the family affluence category. While in the high-family affluence boys category of adolescents, achieving 60 min of MVPA daily and the absence of excessive ST on weekdays significantly reduced their odds of being overweight/obese, in the low- family affluence adolescents this was not the case.
**Sigmund et al., 2015a** [64]	n = 19,940^d^ 51% girls 10.5–16.5 years Czech Republic	Self-reported (HBSC questionnaire)	PA (MVPA, VPA) SB (ST)	Between the years 2002 and 2014, significant decreases in meeting the MVPA recommendations were evident for both adolescent boys and girls. Moreover, increases in excessive ST on weekdays and at weekends were found in the boys.
**Sigmund et al., 2015b** [85]	n = 485 51% girls 9–12 years Czech Republic	Device-measured (Digi-walker Yamax SW-200 pedometer, 7 consecutive days, >10 valid hours per day, at least 4 weekdays and 2 weekend days)	PA (total, steps per day) SB (ST)	A quantifiable relationship between parent-child steps per day and mothers’ ST and children’s steps at weekends was found. Each 1000-step increase in mothers’ (fathers’) steps per weekend day was associated with an extra 523 steps per day in their daughters and 508 steps per day in their sons. A reduction in mothers’ ST by 30 min per weekend day was associated with an extra 494 steps per day in their daughters and 467 steps per day in their sons.
**Sigmund et al., 2014** [65]	n = 338 50% girls 9–11 years Czech Republic	Device-measured (ActiTrainer accelerometer, waist, 2 consecutive days, >12 valid hours per day, 15-s epoch)	PA (LPA, MVPA) SB (total sedentary time)	Participation in PE lessons led to higher school and daily MVPA in the overweight/obese and normal-weight girls and boys. Participation in PE lessons also reduced school-time SB in the overweight/obese children and normal-weight girls.
**Sigmund et al., 2013**^**a**^ [49]	n = 176^d^ 48% girls 10–12 years Olomouc region	Device-measured (Digi-walker Yamax SW-200 pedometer, hip-worn, 7 consecutive days, >12 valid hours per day)	PA (steps per day)	The study indicated favourable effects of a daily school-based PA intervention programme on the lower incidence of overweight/obesity, which was maintained two years after the end of the direct involvement of the researchers.
**Sigmund et al., 2012**^**c**^ [50]	n = 176 48% girls 6–9 years Olomouc region	Device-measured (Caltrac accelerometer, Digi-walker Yamax SW-200 pedometer, hip-worn, 7 consecutive days, >8 valid hours per day)	PA (steps per day)	School-based PA (PE lessons, PA during short breaks and longer recesses, PA at the after-school nursery) in compatible active environments (child-friendly gym and school playground, corridors with movement and playing around corners and for games) had a vital role in reducing obesity and overweight among younger pupils.
**Sigmund et al., 2011**^**c**^ [51]	n = 176 48% girls 6–8 years Olomouc region	Device-measured (Caltrac accelerometer, Digi-walker Yamax SW-200 pedometer; hip-worn, 7 consecutive days, >8 valid hours per day)	PA (steps per day)	Higher school PA significantly enhances the inhibition of the decline in daily PA and the increase of obesity in 6-8-year-old children. After two years of the intervention programme, there was no occurrence of obesity in any child in the experimental group, and yet 22% of the girls and 23% of the boys in the control group were obese.
**Sigmund et al., 2009**^**a**^ [100]	n = 176 48% girls 5–7 years Czech Republic	Device-measured (Caltrac accelerometer, Digi-walker Yamax SW-200 pedometer; hip-worn, 7 consecutive days, >8 valid hours per day)	PA (steps per day)	The first-grade schoolchildren had lower PA than the children attending pre-school on weekdays and at weekends. There was a decline in PA on weekdays during time spent at school and not during the children’s after-school leisure time.
**Sigmund et al., 2008** [42]	n = 193 44% girls 8–13 years Olomouc region	Self-reported (IPAQ-SF)	PA (total, OLTA, VPA, MPA, walking) SB (sitting time)	A longer duration of total PA in daughters, sons, and their fathers and mothers was related to a shorter daily period of time spent sitting. A longer time spent sitting by parents daily was associated with a longer time spent sitting by their children. Children, both daughters and sons, and their mothers who participate in OLTA twice or more times a week showed a significantly longer time spent performing VPA than children whose mothers are without any participation in OLTA.
**Sigmund et al., 2007** [43]	n = 67 48% girls 9–11 years Olomouc region	Device-measured (Caltrac accelerometer, Digi-walker Yamax SW-200 pedometer, 7 consecutive days)	PA (steps per day)	More than 73% of the participating children who were physically active at the weekend at least at the same level as on school days met the health-related PA recommendations for this age category. The weekly number of steps for children who met the health recommendations was 6000 steps per day higher than for the children who did not meet the health recommendations.
**Sigmundová et al., 2020a** [95]	n = 1795 4–16 years Czech Republic	Device-measured (Digi-walker Yamax SW-200 pedometer; hip-worn, 7 consecutive days, < 8 valid hours per day)	PA (steps per day)	A strong parent-child step count relationship was found in the children younger than eight years of age. In the older children, the parent-child step count association was gender-specific and dominated by the father-son relationship, particularly at weekends.
**Sigmundová et al., 2020b** [102]	n = 1284 51% girls 4–16 years Czech Republic	Device-measured (Digi-walker Yamax SW-200 pedometer, 8 consecutive days)	PA (steps per day, OLTA) SB (ST)	Despite the different mother-/father-child behavioural associations, the daily step counts of the parents were positively associated with the daily step counts of their children. For both overweight/obese and non-overweight children, the odds of reaching the recommended daily step counts were increased by their regular participation (≥twice per week) in OLTA and non-excessive entertainment ST (≤2 h per day) in the mother-child and nuclear family triads.
**Sigmundová et al., 2018** [72]	n = 649^d^ 51% girls 7–11 years Czech Republic	Device-measured (Digi-walker Yamax SW-200 pedometer; hip-worn, 8 consecutive days, >8 valid hours per day, at least 4 weekdays and 2 weekend days)	PA (steps per day) SB (ST)	High levels of parents’ PA contributed to the achievement of the recommended daily PA in children on weekdays and at weekends. Excessive weekend ST of parents reduced the odds of their children achieving the recommended daily PA; however, the influence of parents’ PA on their children’s achieving the recommended daily PA was stronger than the inhibitory effect of ST.
**Sigmundová et al., 2017** [84]	n = 16,535^d^ 51% girls 11–15 years Czech Republic	Self-reported (HBSC questionnaire)	SB (ST)	The boys and girls surveyed in 2014 were up to two times more likely to meet the recommendations for watching television in comparison with groups of schoolchildren of the same age surveyed in 2002. In contrast, computer use by adolescents increased markedly between 2006 and 2014. Taking total ST into account, spending two hours per day or less on it decreased significantly among boys and girls between 2006 and 2014.
**Sigmundová et al., 2014** [73]	n = 485 51% girls 9–12 years Czech Republic	Device-measured (Digi-walker Yamax SW-200 pedometer; 7 consecutive days, >10 valid hours per day)	PA (steps per day) SB (ST)	The children of fathers and mothers who met the weekend recommendation of 10,000 steps were 5.48 and 3.60 times respectively more likely to achieve the weekend recommendation than the children of less active parents. The children of mothers who reached the weekday pedometer-based step count recommendation were 4.94 times more likely to fulfil the step count recommendation on weekdays than the children of less active mothers.
**Sigmundová et al., 2013** [36]	n = 14,219^d^ 51% girls 11–15 years Czech Republic	Self-reported (HBSC questionnaire)	PA (MVPA) SB (ST)	In comparison with 2002, increased sedentary time and a decline or stagnation of the proportion of children meeting the recommendations for PA were found among Czech school-aged children in 2010.
**Sigmundová et al., 2011** [33]	n = 520 60% girls 14–18 years Czech Republic	Device-measured (Digi-walker Yamax SW-701 or Omron HJ-105 pedometer, hip-worn, 7 consecutive days, >10 valid hours per day)	PA (total, steps per day) SB (total sedentary time)	A secular decrease in PA was found amongst adolescents between 1998–2000 and 2008–2010. The significant interaction effects (cohort × age; and cohort × gender) that this study found suggested that secular trends in PA differ by age and gender.
**Šimůnek et al., 2017** [66]	n = 701 60% girls Mean age: 17.0 years Czech Republic	Self-reported (IPAQ-LF)	PA (total, AT, LPA, MVPA) SB (sitting time)	The differences in the overall weekly PA between boys and girls attending secondary school and university were significant, with the boys being more active than the girls in both cases.
**Šnoblová et al., 2015** [94]	n = 169 50% girls 9–10 years Czech Republic	Device-measured (ActiTrainer accelerometer, hip-worn, 2 days, >12 valid hours per day, 15-s epoch)	PA (total, steps per day, MVPA)	The boys were more physically active than the girls during their time at school, in terms of both their step count (800 steps per day more than girls) and the duration of MVPA.
**Valach et al., 2017** [56]	n = 653 59% girls Mean age: 17.3 years Plzeň region	Self-reported (IPAQ-LF, questionnaire – Sport Preferences Survey)	PA (total, MPA, VPA)	A preference for fitness activities was associated with a higher level of PA in the spare time of boys, and with the VPA of the boys and girls, compared to those who did not prefer these activities. In addition, in the case of the boys, significant correlations were found between a preference for team sports and PA at school. Individual sports (swimming, cycling, and downhill skiing) are the main PA preferred by girls. These activities were followed by team sports and rhythmic and dance activities. In the case of the boys, team sports (football, floorball, and basketball), individual sports, and fitness activities appear in the top positions.
**Valová et al., 2013** [38]	n = 55 100% girls Sixth-grade class Opava region	Device-measured (ActiGraph GT3X accelerometer, hip-worn, 7 consecutive days)	PA (total, coordination skills)	There was no difference in PA between the girls from cities and those from villages.
**Vašíčková et al., 2013** [90]	n = 786 66% girls 15–16 years Czech Republic^b^	Device-measured (Digi-walker Yamax SW-700 pedometer) Self-reported (IPAQ-LF)	PA (steps per day)	On average, young people recorded lower numbers of steps at weekends compared to schooldays, with Sunday being the most critical day of the week. No significant differences were found between the boys and girls in terms of the average number of steps per day.
**Vašíčková et al., 2008**^**a**^ [39]	n = 9 100% girls Mean age: 15.6 years Olomouc region	Device-measured (Omron HJ-105 pedometer, hip-worn, 10 months, >10 valid hours per day)	PA (steps per day)	Variability in year-round PA was found across days and the autumn, winter, and spring months. Saturdays and Sundays were the days with the lowest numbers of steps. The highest number of steps was achieved on days with PE lessons. The months with the lowest and highest average numbers of steps per day were February and June, respectively.
**Vindiš et al., 2019**^**a**^ [40]	n = 27 100% girls 11–15 years Czech Republic	Device-measured (ActiGraph GT3X+ accelerometer, hip-worn, 7 consecutive days, 60-s epoch, >13 valid hours per day, at least 3 weekdays and 1 weekend day, CoP by Evenson)	PA (total, LPA, MPA, VPA) SB (total sedentary time)	The volume of SB and PA was different on the days with training, on the days without training, and during the seasons. On the days with PE lessons, the girls had 27.6% more MPA, 37.7% more VPA, and 8% fewer SB than on the days without PE lessons.
**Vorlíček et al., 2020** [88]	n = 1586 48% girls 11–15 years Czech Republic	Self-reported (HBSC questionnaire and IPEN Adolescent questionnaire)	PA (AT – commuting to/from school)	Most of the Czech adolescents misperceived the active commuting norms of their peers. 68% of the Czech adolescents in this study were daily active commuters (walking, cycling, or riding a scooter or skateboard).
**Vorlíček et al., 2019** [67]	n = 1586 48% girls 11–15 years Czech Republic	Self-reported (Czech version of the questionnaire from the SONIAA study, Youth Activity Profile)	PA (MVPA on Saturday)	The level of the real PA of an individual and the perceived level of Saturday’s PA in peers differed significantly among Czech adolescents. The pupils believed that 41.9% of their classmates had had at least one hour of PA on Saturday. This estimation differs by 18% points from the reported situation.
**Vorlíček et al., 2017** [74]	n = 1745 49% girls 11–19 years Czech Republic	Device-measured (Digi-walker Yamax SW-700 pedometer, 7 consecutive days) Self-reported (IPEN Adolescent questionnaire)	PA (AT – commuting to/from school, steps per day)	A greater proportion of pupils who met the health recommendations for PA occurs in the group using active modes of commuting to school, such as walking or riding a bike or skateboard. The majority (85%) of the pupils who commuted actively to school lived within a 20-minute walking distance.
**Weinberg et al., 2019** [96]	n = 4847^d^ 11–15 years Czech Republic^b^	Self-reported (HBSC questionnaire)	PA (MVPA, VPA)	Daily involvement in MVPA decreased with age (from 4.62 days with MVPA per week in the 11-year-old adolescents to 3.99 days with MVPA per week in the 15-year-old adolescents) while weekly VPA increased (from 1.99 h per week in the 11-year-olds to 2.52 h per week in the 15-year-old adolescents).
**Whiting et al., 2020** [97]	n = 1406^d^ 52% girls 6–9 years Czech Republic^b^	Self-reported (questionnaire from the COSI)	PA (active play, OLTA, AT – commuting to/from school) SB (ST)	Approximately 98% of the Czech children play actively for at least 1 h per day; 37% were not members of sports/dance clubs or did not do sports or dance at all. Approximately 45% of the Czech children used AT (walking or cycling) to get to and from school, 64% of the children spent <2 h on ST per day, and 95% of the children slept for 9–11 h per night.

PA – Physical activity, SB – Sedentary behaviour, ST – screen time, AT – Active transportation, PI – Physical inactivity, PE – Physical education, MVPA – Moderate to vigorous physical activity, MPA – Moderate physical activity, VPA – Vigorous physical activity, LPA – Light physical activity, BMI – Body mass index, OLTA – Organized leisure-time activities, HBSC – Health Behaviour in School-Aged Children, IPAQ-LF – International Physical Activity Questionnaire – long form, IPAQ-SF – International Physical Activity Questionnaire – short form, IPEN – International Physical Activity and the Environment Network, QPAP – Questionnaire on Physical Activity Preferences, SONIAA – Social Norms Intervention for Active

Adolescents, COSI – Childhood Obesity Surveillance Initiative

^a^Longitudinal study

^b^International study

^c^Intervention study

^d^Representative sample

^e^*Note*: Kalman et al., 2015b [30]: These were trend analyses in the implementation of recommendations for the 32 states participating in the HBSC from 2002, 2006, and 2010. Sample size data is not reported for the individual studies

Sixty-one articles were published in the English language and 18 were published in the Czech language. Out of 79 articles, 18 papers [23–32, 35, 46, 57, 68, 79, 90, 96, 97] were part of international surveys with clearly identified results related to PA and/or SB in Czech children and adolescents.

**Fig. 2:**
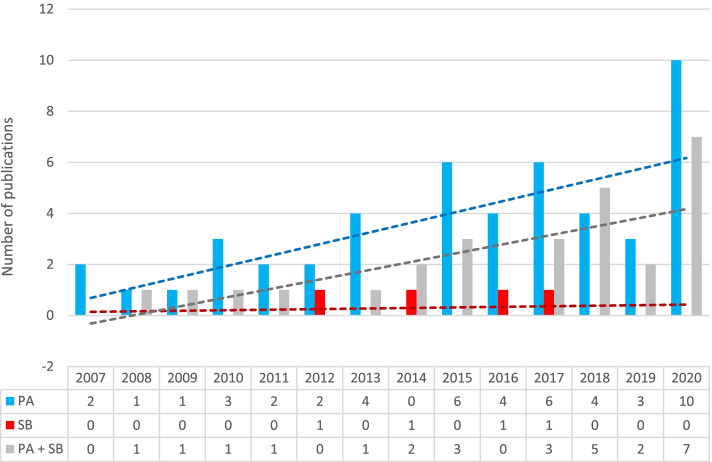
An overview of the studies included considering their year of publication and primary outcomes. *Note.* PA= Physical activity; SB = Sedentary behaviour

An apparent increasing trend of published articles is shown in Fig. 2. The first two papers related to the PA of Czech children and adolescents were published in 2007 [43, 71]. In addition, there are three papers published later on for which the data collection took place in 1998–2000 [33], 2001 [34], and 2002 [36], and thus the data related to the PA or SB of Czech children and adolescents in the identified articles covers the whole 20-year period.

### Descriptive characteristics of the available evidence

#### Study purposes

The main purposes of the selected articles were mainly the description of: (1) PA [27, 38, 43, 45, 47, 48, 56, 66, 68, 71, 74, 94, 99] or SB [27, 55, 66]; (2) the prevalence of sufficient PA [46, 54, 55, 90] or excessive SB [79, 80, 83], and (3) trends or changes in PA [26, 30, 33, 34, 36, 60, 63, 64, 86, 100] and SB [33, 36, 84]. The main aims of further articles were the examination of the relationships between PA or SB and (1) school settings and school-related factors [25, 28, 32, 57, 69, 87, 89, 92, 101]; (2) behavioural and psychosocial factors (e.g. health-related behaviours [29, 44, 81, 89, 91–93], psychological factors [24, 46, 56, 59, 67, 69, 75, 88], or social and family factors [42, 61, 72, 73, 76–78, 82, 85, 95, 101]; 3) the social and built environment [23, 35, 70, 96, 98] and seasonal changes [37, 39, 40], and 4) obesity-related indicators [41, 44, 52, 53, 55, 58, 65]. In addition, several articles investigated the influence of school PA programmes on the incidence of obesity [49–51].

#### Study sample

This scoping review identified 70 cross-sectional articles, seven longitudinal articles, and two interventions. Out of all the cross-sectional articles including school-aged participants, 18 articles focused on children, 54 articles on adolescents, and seven articles on both. The ages of the study participants ranged between six and 20 years. The intervention studies employed only children aged from six to nine years. Girls represented more than 50% of the study sample in most of the articles and boys were not included in four articles [37–40]. Out of all 79 articles, randomly selected representative samples were identified in 28 articles.

In the cross-sectional articles, the sample size varied from 55 [38] up to 30,966 participants [41]. The longitudinal articles employed samples with from nine to 176 participants.

This scoping review identified data on the PA and SB of Czech children and adolescents in 18 international and 61 national articles. Some of the national articles narrowed their selection only to specific regions: 12 articles from the Olomouc region [34, 39, 42–45, 47–51, 79], two articles from the Hradec Králové region [34, 45], three articles from the Moravia region [37, 52, 53], two articles from the North-western Bohemia region [54, 55], and one article from each of the Plzeň [56] and Opava regions [38].

#### Measurement

**Fig. 3:**
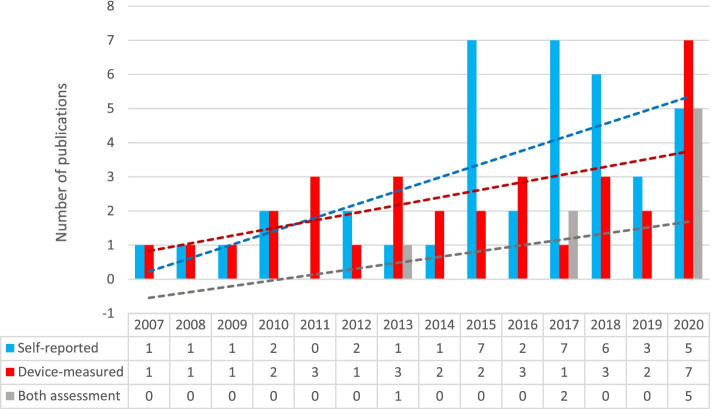
An overview of the studies included, considering their year of publication and the assessment tools

None of the articles used qualitative research methods. All of them used quantitative methods and reported the measurement tool for the assessment of PA or SB (Table 1). The number of articles using self-reported (*n* = 39), device-measured (*n* = 32), and a combination of both (*n* = 8) assessments of PA and SB is shown in Fig. 3. Out of 40 articles using device-based measurements, accelerometers (ActiGraph GT3X, GT3X+, GT9X Link, wGT3X-BT, Caltrac, and ActiTrainer) were used in 19 articles, while pedometers (Digi-walker Yamax SW-200, SW-700, SW-701, Omron HJ-105) were used in 28 articles. Studies using self-reported measurements used HBSC questionnaires (*n* = 27), IPAQ long form (*n* = 13), and International Physical Activity and the Environment Network (IPEN) Adolescent questionnaires (*n* = 2), IPAQ-short form (*n* = 1), and other types of questionnaires (*n* = 9).

Of the 79 articles included (Fig. 2), PA-only articles comprised the largest proportion (61%), followed by articles of both PA and SB (34%) and SB-only articles (5%). PA was expressed as minutes, MET-minutes, or steps on an average day (waking time or 24 h) or a specific segment of the day (unorganized or organized leisure-time physical activity (OLTA), school PA in physical education (PE) lessons, or, during recesses, active transportation (AT). The SB articles reported minutes of total SB, sitting time, or ST, which was defined as TV viewing, playing games on a PC/console, chatting online, using the internet, emailing, etc.

### Main findings related to PA and SB

#### Prevalence of sufficient/insufficient PA and SB

Thirty-seven articles (Supplementary file 3) also reported the prevalence of sufficient/insufficient PA, SB, or both. The prevalence of the adherence to the PA and ST recommendations ranges between 12.6 and 73% and 17.5 and 73.6%, respectively. However, the definition of a sufficient PA level was different in the articles that were included, ranging from achieving 60 min of moderate-to-vigorous physical activity (MVPA) daily or five times a week [23–26, 30, 32, 46, 49, 53, 58–67, 69], 20 min of VPA at least three times a week or 30 min of MPA or walking at least five times a week [24–26, 32, 46, 56, 59, 64, 66, 70, 71], and daily step counts ranging between 11,000 and 14,000 for boys and 10,000 and 13,000 for girls [26, 33, 43, 60, 69, 72–78, 90]. Moreover, the recommendation for school PA (i.e. 500 steps/school time) was met by 39% of Czech adolescents [28] and 83% of boys and 69% of girls on days with a PE lesson [69]. Excess SB was mostly defined as ≥ 2 h per day of ST [64, 72, 73, 80] and was accomplished by 40.4–80.7% of children and adolescents.

The correlates of meeting the PA and SB recommendations were examined in 28 [23–26, 28, 30, 32, 33, 36, 43, 46, 53, 56, 59–62, 65–67, 69–71, 74, 75, 77, 78, 90] and three articles [80, 84, 97], respectively. Six articles [58, 63, 64, 72, 73, 76] examined the correlates of meeting both the PA and SB recommendations. A greater likelihood of achieving the recommended level of PA was associated with lower age [32, 36, 37, 57–59, 62, 64, 67, 71, 81, 96], an environment perceived as activity-friendly [35], participation in PE lessons or intervention programmes [50, 65, 69], active transportation [24, 67], or OLTA [71, 77, 78, 82], or more specifically in sports clubs [32], sufficient PA on weekend days [73, 90], preferences for fitness activities [46, 56], different kinds of motives [26, 38, 44, 45, 59], and the agreement between preferred PA and that actually undertaken [26].

The proportion of children and adolescents not meeting the recommendation for SB or ST was positively associated with age [58, 79, 80, 83, 84] and more ST on weekend days compared to weekdays [27, 36, 55, 64, 73, 80, 84, 85]. Furthermore, longitudinal studies confirmed the variability of the achievement of sufficient PA and SB across the seasons, months, and days of the week [37, 39, 40, 47, 48].

Ten articles investigated the trends of sufficient PA or SB and found an increasing prevalence of sedentary time [36, 64] and a decreasing prevalence of children and adolescents meeting PA guidelines [26, 30, 33, 46, 60, 63, 64] and using active modes of travel when commuting [86]. However, two articles suggested that the way children and adolescents spent their time being sedentary had shifted from watching TV to computer use between 2002 and 2014 [63, 84]. Moreover, an increasing prevalence of SB and a decreasing prevalence of achievement of the PA recommendations in adolescents were in line with a concurrent increase in the prevalence of their overweight/obesity [36, 64].

#### Weekdays versus weekend days

In all the articles, a greater PA level was found for weekdays compared to weekend days. Moreover, this was confirmed in longitudinal studies [39, 48] which revealed that across all months and seasons, children and adolescents achieved notably more minutes in MVPA or steps on weekdays than at weekends, with Sunday being the least active day [39, 47, 48, 60]. Conversely, weekend days seem to be the “risk time” for excessive ST and insufficient PA levels [36, 60, 64, 73, 80, 85].

#### Active transportation

Out of 11 articles examining AT, nine focused on active commuting to and from school, including walking, cycling, and riding a scooter or skateboard. The proportion of children and adolescents using actively commuting to school ranged between 22.5% and 74.3% across the studies [31, 34, 61, 74, 86–88, 97, 101]. Living within a 20-minute walking distance to school [74], a place of residence being in the same municipality as the school [61, 101], high-walkable areas [34], and attending schools with policies and programmes promoting AT [87, 101] were positively associated with active commuting to school. According to one article [24], AT accounted for 22.5% and 24.9% of the weekly PA of Czech boys and girls, respectively. A long-term decrease in the AT of Czech adolescents was found in two articles. One article [34] found a decrease in the proportion of active commuting in 47% of the respondents and suggested a decrease of 2.7 times in the likelihood of commuting actively in 2011 in comparison to 2001. The second articles [86] showed an overall decrease in AT by 21.7% in boys and by 23% in girls between 2006 and 2014.

#### PE lessons and organized leisure-time activities

Participation in regular PE lessons contributed considerably to an increase in the volume and intensity of school PA [28, 65, 69], steps per day [39, 48], and daily MVPA [65], and a decrease in school-time SB [65]. This was apparent across all months and seasons [39, 40, 48]. In intervention study indicating the effect of school-based PA programmes on the incidence of obesity [49–51], PE lessons as a part of the school PA programme performed in compatible active environments were found to have a vital role in the reduction of obesity and overweight among younger pupils.

The proportion of children and adolescents who participated in OLTA ranged between 41 and 81% across articles [89, 91–93] and decreased with age [55, 91]. More specifically, 62% of Czech children and adolescents took part in sports club activities, according to [32]. In all 14 articles [32, 42, 47, 55, 71, 76, 77, 82, 89, 91–93, 97, 102] that investigated OLTA, only positive effects of this activity were suggested. Specifically, OLTA was positively associated with better physical and mental health [93], a lower rate of occurrence of repeated substance use and truancy, and inversely with higher odds of physical fights and injuries [91], higher school engagement, lower levels of school-related stress, and better academic achievement [92], and a lower prevalence of obesity [82, 85]. In addition, children with regular OLTA were more likely to meet the PA recommendations [32, 55, 77, 82, 102], having higher levels of VPA and spending less time sitting than children without participation in OLTA [42]. Longitudinal engagement in OLTA increased daily PA, regardless of the month and season [47]. In contrast, 34–37% of Czech children and adolescents were not engaged in any OLTA [89, 97]. However, involvement in peer-oriented unstructured activities was strongly associated with an increased risk of smoking, getting drunk, experience with sexual intercourse, and poorer academic achievement in 34% of adolescents [89].

#### Differences between boys and girls

The majority of articles investigating PA in both sexes suggested that boys are more active than girls [53, 71, 94]. This was obvious particularly in total PA [27, 32, 71], MVPA [25, 26, 30, 36, 52, 53, 57, 59, 60, 63–65, 94], school-based PA [27, 28, 69], leisure-time PA [55, 71, 82, 89, 92, 93], and active transportation [24, 34, 86, 87]. Although most articles found a higher daily number of steps in boys than in girls [26, 28, 49–51, 53, 54, 60, 68, 69, 72, 73, 82, 85, 94, 95, 102], four articles conversely suggested more steps per day in girls compared to boys [27, 33, 75, 77]. Sex-specific patterns related to PA were found in several articles. For example, boys were more likely to cycle to school [86] and the association between school policies and programmes and AT was stronger in boys compared to girls [80]. On the other hand, a higher rate of AT was significantly associated only with greater well-being in girls [24]. Girls had significantly lower odds of adherence to the combined movement guidelines [62] and appeared to be more influenced by social motives [59] and their participation in leisure-time PA decreased with age more than was the case for boys [55]. PA preferences differed between boys and girls and were longitudinally more stable for boys than for girls [46].

The gender differences were obvious in the relationship between PA or SB and obesity indicators. MVPA was negatively associated with body fatness indicators only in girls, while body fatness indicators in boys were strongly negatively associated only with vigorous PA [53].

In SB articles, the results related to sex differences were not so clear. In some device-based articles, girls were found to be more sedentary [33, 53, 65, 70], while in others, the time spent on SB was greater in boys compared to girls [41, 55, 63, 80, 83]. Moreover, in self-reported articles, boys reported higher overall ST [55, 83], computer use [27, 41, 80], or TV watching [41] than girls. On the contrary, chatting online was more common among girls [80]. Sex-specific SB was also found in a device-based article [52], where boys spent on average 9.9 min per day less on short sedentary bouts and 7.5 min per day more on long sedentary bouts compared with girls. Also, longitudinal increases in excessive ST on weekdays and at weekends were found only in boys [64]. Additionally, watching TV was positively associated with being overweight/obese only in adolescent girls, but not in boys [41].

#### Parent-child PA

The relationship between parentsʼ and childrenʼs PA and SB was investigated in eight articles [42, 72, 73, 77, 82, 85, 95, 102]. In conclusion, high levels of parental PA contributed to the achievement of the recommended daily PA in children [72, 73, 77, 85, 95, 102], and excessive ST of parents was associated with more time spent sitting in their children [42] and a reduced likelihood of their children achieving the recommended daily PA [72, 85]. The parent-child PA or SB relationship was found to be stronger for children younger than eight years of age [77, 95], on weekend days [72, 73, 85, 95], and in families participating in organized PA [42]. In addition, sufficient PA in mothers was associated with sufficient PA in their overweight/obese children [77].

#### 24 h time use

In four articles, 24-hour movement behaviours were considered, including SB, PA, and sleep. On the evidence of one self-reported study [97], 95% of Czech children slept for nine to 11 h per night. Approximately three-quarters of children (71%) and adolescents (75.3%) slept for less than nine and eight hours, respectively [81]. In children, this short duration of sleep was associated with higher SB by 95 min per day and lower MVPA by 16 min per day. In adolescents, a higher SB by 67 min per day and lower light-intensity PA (LPA) by 2 min per day were found to be associated with a short duration of sleep [81]. Also, meeting the combined 24 h movement guidelines (≥ 60 min per day of MVPA, < 2 h per day of recreational ST, and uninterrupted sleep for 9–11 h per day for children or 8–10 h per day for adolescents) was investigated in two studies [58, 62]. All the recommendations of the combined 24 h movement guidelines were met by approximately 6.5% of the children and 2.2% of the adolescents [62]. While no associations were found between meeting all three (combined) recommendations and adiposity indicators, meeting only the ST recommendation or the combined ST and sleep recommendations was found to be negatively (favourably) associated with adiposity [58]. Furthermore, significantly lower odds of adherence to the combined movement guidelines were found in girls and participants with overweight or obese fathers [62].

## Discussion

This scoping review examined 79 articles related to the PA or SB of Czech children and adolescents and summarized the descriptive characteristics and main conclusions of the available evidence. The majority of the articles were cross-sectional (88.6%), approximately two-thirds of the articles (61%) examined only PA, and half of the articles (50.6%) employed device-based assessments. Approximately 47% of the articles reported the prevalence of sufficient/insufficient PA and 14%, 23%, and 10% of studies focused on AT, organized PA (including PE or OLTA), and parent-child PA, respectively.

Although we searched for articles published in the past two decades, the articles included in this review were not published until 2007. Since then, the amount of PA and SB research has been on the rise, with 79 articles being published till December 2020. This is in line with the gradual increase in the number of Czech studies dealing with the PA of children and adolescents, often connected with international cooperation and participation in global studies, such as International Physical Activity and the Environment Network, Health Behaviour in School-Aged Children, or the Childhood Obesity Surveillance Initiative. Although the first two papers were published in 2007, data from several of the articles that were identified was collected much earlier. This is mainly apparent in trend articles, with the first round of assessments in 1998–2000 [33], 2001 [34], and 2002 [36]. Hence, the conclusions from selected articles reflect a more than 20-year period of the assessment of Czech children’s and adolescents’ PA.

 In this review, we found a high proportion of cross-sectional articles and only nine articles with a longitudinal design. The follow-up periods of the longitudinal studies ranged between ten and 24 months and revealed differences across days of the week, months, and seasons, and in one specific life transition (i.e. from kindergarten to school). However, these studies mainly employed very small convenience samples, with a limited possibility of generalizing the results to the entire Czech school-aged population. Considering longitudinal studies as the way to define the causality and determinants of PA and SB, more longitudinally designed studies with a random sample are needed in the Czech Republic. Additionally, these studies should consider the year-round movement behaviours concerning different life transitions.

This study critically reviewed only the PA and SB research in the target populations, although the importance of sleep has been recognised and was included as an important health outcome when considering the impact of PA and SB [81]. This was done for two reasons. Firstly, the vast majority of Czech papers focused only on PA and/or SB, with the exception of four papers [52, 58, 62, 81] published very recently which examined movement behaviours (i.e. PA, SB, and sleep) using a 24-h wear time protocol. Secondly, this approach agrees with a recently published edition of the “WHO Guidelines on Physical Activity and Sedentary Behaviour” [1]. However, in recent years, the univariate paradigm in movement behaviour research has been replaced with a 24-h time-use paradigm that integrates the analysis of all daily movement behaviours relative to each other, rather than as individual entities [103]. This approach is apparent in world initiatives, such as developing time-use epidemiology [104] and in the creation of 24-h movement behaviour recommendations in several countries, such as Australia [105], Canada [106], Croatia [107], New Zealand [108], or Thailand [109]. In this review, the presence of studies covering all daily behaviours is a promise of the quality of the PA-related research that could be the core for developing a comprehensive surveillance system in the Czech Republic.

Consistent, valid, and reliable assessments of prevalence and trends in PA (ideally relative to other behaviours) are necessary to guide the development of policies and programmes to increase activity levels and to reduce the burden of non-communicable diseases associated with physical inactivity and elevated SB. In this review, 37 articles investigating the percentage of Czech children or adolescents who achieved the PA or SB recommendations were cross-sectional, with 62% not having a representative sample.

This review also identified several papers that examined trends in PA or SB or meeting the recommendations for these behaviours. Such studies with randomly selected samples mirrored the negative trends which were found in other countries [5], such as increased sedentary time, a decreasing percentage of children and adolescents meeting the PA guidelines, and using AT. However, these studies employed self-reported assessments of PA and SB that could be prone to both inaccurate reporting and reporting bias [110].

In this review, approximately half of the articles used self-reported assessments of PA or SB and half of them device-based ones. Among the device-based studies, pedometers have been widely used in the last twenty years in the Czech studies. However, the feasibility of using pedometers in a state-based surveillance system were modest at best [111]. Accelerometers seemed to be more accurate in the characterization of the entire activity pattern in school-aged children [112]. On the other hand, the domain- and type-specific differences in PA and SB (e.g. AT, ST) advocate the importance of assessing movement behaviours using a combination of self-reported and device-based methods. However, only eight articles were found to use this combination of methods and hence, an increase in the number of such studies in the future could be a possible direction for improving the surveillance system in the Czech Republic.

Furthermore, the variety of tools used to assess PA and SB and the way in which the data was processed (especially in accelerometers), as well as the different definitions of sufficient levels of PA and SB in the studies, have to be mentioned and may explain the inconsistency of findings related to sex-specific PA and SB or the prevalence of sufficient PA.

### Strengths and limitations of the study

 This is the first comprehensive study that systematically reviews the available evidence related to PA and SB in Czech children and youth. Moreover, within this review, we provide suggestions for future directions of the national surveillance system on these behaviours.

Several limitations have to be mentioned. Firstly, the search strategy does not cover “grey literature”. This could lead to publication bias as we might have missed some valuable data published in conference proceedings, Master’s or doctoral theses, or reports. On the other hand, our search strategy ensured that all the studies found for this review underwent a peer review process and demonstrated at least satisfactory methodological quality standards. Secondly, within this scoping review we described the study designs, samples, and methods of PA and SB assessments and the main findings of the selected studies. However, an assessment of methodological quality which could help in creating a comparison of the outcomes of the studies was not carried out. Last but not least, the main findings presented in this review were summarized from 79 individual articles referring to 27 original studies and using different tools, study samples, and definitions of PA and SB. The interpretation of these findings and their implications should be used with caution.

## Conclusion and recommendations

 This scoping review identified 79 articles from 27 studies related to PA and SB in Czech children and adolescents and revealed an increasing trend in the number of articles published during the past two decades. The results of studies suggested an increase in sedentary time or its proxy indicators and a decreasing percentage of Czech children and adolescents doing a sufficient amount of PA. On the evidence of this review, organized PA, including PE lessons as well as OLTA, increasing weekend PA, family inclusion, and an environment that supports movement might contribute to a reduction of unhealthy time use in Czech children and adolescents.

Although we reviewed 79 articles, standardized national surveillance was not recognised. This review identified limitations, including the large proportion of cross-sectional studies, the limited sample size in longitudinal studies, and the lack of studies using a mix of device-based and self-reported methods and focusing on health-related 24-h time use patterns.

In agreement with global calls [11, 113], the result of this review advocates the necessity of the government-supported development of a national surveillance system that would incorporate a combination of standardized device-based measures, such as those derived from accelerometers, and self-reported domain and type-specific assessments. Such a national surveillance system on movement behaviours, which would allow the recommended amount of these behaviours to be systematically assessed, is needed to reduce the decrease in sufficient PA and hence, improve the health of Czech children and adolescents or reduce the risk of different non-communicable diseases in this group.

With respect to the “WHO Global action plan on physical activity 2018–2030” call [11] and on the basis of this review of the available evidence, we identified several issues which might serve as a recommendation for future directions in Czech research and policy and be inspiring for other Central European countries lacking a national surveillance system:

- We advocate the development of a national surveillance system on 24-h movement behaviours that would incorporate both device-based measures and self-reported domain and type-specific assessments.
- The national surveillance system should systematically monitor the prevalence of sufficient PA and 24-h time use of PA, SB, and sleep and their associations with health outcomes.
- Further evidence is needed to support national recommendations on PA, SB, and sleep and to enforce them in state and school policy and practice. Furthermore, longitudinally designed studies using both device-based measures and self-reported assessments and employing randomly selected samples of Czech children and adolescents are needed.

## Supplementary Information

**Additional file 1.**

**Additional file 2.**

**Additional file 3.**

## Data Availability

The data supporting the conclusion of this paper is available through the detailed reference list. No original datasets are presented since this was a review of previously existing literature. Data sharing is not applicable to this article as no datasets were generated or analysed during the current study.

## Abbreviations

PA: physical activity
SB: sedentary behaviour
METs: metabolic equivalents
WHO: World Health Organization
OLTA: organized leisure-time physical activity
PE: physical education
AT: active transportation
ST: screen time
MVPA: moderate-to-vigorous physical activity
LPA: light-intensity physical activity
