# Supplementary material for: Surveillance of physical activity and sedentary behaviour in czech children and adolescents: a scoping review of the literature from the past two decades
Source: BMC Public Health. 2022 Feb 21;22:363. doi: 10.1186/s12889-022-12766-0 (PMC8859875; doi:10.1186/s12889-022-12766-0)
Supplement: Supplementary file 2 — Additional file 2. [file 12889_2022_12766_MOESM2_ESM.pdf]

Search strategy for the Medline database (via OVID)

1. exp Exercise/

2. *Motor activity/

3. *Leisure Activities/

4. *Healthy Lifestyle/

5. *Locomotion/

6. ((light or moderate or vigorous or "moderate to vigorous" or "moderate-to-vigorous") and (intensity or "physical activity" or PA)).tw

7. LPA or LIPA or MPA or MVPA or VPA.tw

8. ((physical activit* or movement) adj3 (recommendation* or guideline*)).tw

9. "active lifestyle" or "physical activity".tw

10. exp Accelerometry/

11. accelerometer or pedometer or steps or "step count" or "steps per day" or "actigraphy".tw

12. (fitness or consumer-wearable or activity) and tracker*.tw

13. "physical activity questionnair*" or " IPAQ" or " international physical activity questionnaire" or "GPAQ" or "global physical activity questionnaire".tw

14. exp Play and Playthings/

15. exp Sports/

16. ((organized or participation or preference or individual or team) adj3 sport*).tw

17. *Transportation/

18. "active transport" or "cycling" or "walking" or "roller skating".tw

19. *"Physical Education and Training"/

20. ("before school" or "in school" or "after school" or "school-based" or "school based") and ("physical activit*" or PA).tw

21. ("physical activity" or PA or sedentary) adj3 (break* or bout*).tw

22. 1 or 2 or 3 or 4 or 5 or 6 or 7 or 8 or 9 or 10 or 11 or 12 or 13 or 14 or 15 or 16 or 17 or 18 or 19 or 20 or 21

23. exp Sedentary Lifestyle/

24. "sedentary behavio?r*" or " physical inactivity" or "screen time" or "screen-time" or "sedentarism" or "sedentary time" or "sitting" or "lying".tw

25. (computer or "video game" or smartphone or tablet or internet) adj3 (time or 'use' or usage).tw

26. 23 or 24 or 25

27. exp Child/

28 exp Adolescent/

29. childhood or adolescence.mp

30. (teen* or "young person" or youth* or school-age or adolescent* or child* or juvenile).mp.

31. 27 or 28 or 29 or 30

32. exp Czech Republic/

33. (moravia* or silesia* or bohemia* or moravian-silesian) and (region or area or location).tw

34. 32 or 33

35. 22 and 26 and 31 and 34
