# Supplementary material for: Surveillance of physical activity and sedentary behaviour in czech children and adolescents: a scoping review of the literature from the past two decades
Source: BMC Public Health. 2022 Feb 21;22:363. doi: 10.1186/s12889-022-12766-0 (PMC8859875; doi:10.1186/s12889-022-12766-0)
Supplement: Supplementary file 3 — Additional file 3. [file 12889_2022_12766_MOESM3_ESM.pdf]

The summary of studies reported the prevalence of meeting recommended level of PA and SB.

First author and year of publication	The criterion for recommended level of PA and/or excessive SB	Meeting recommended level of PA and/or SB (%)
Bucksch et al., 2019 [23]	MVPA (7 times 60 min per day)	21.5% of adolescents
Frömel et al., 2020a [24]	MVPA (≥ 5 times 60 min per day)<br>VPA (≥ 3 times 20 min per day)<br>Active travel recommendation (≥ 5 times 30 min per day)	49% girls and 68% boys
Frömel et al., 2020b [75]	Daily step count (11 000 steps per day)	50.0% of boys and 55.1% of girls
Frömel et al., 2018 [25]	VPA (3 times 20 min per day)<br>MPA (5 times 30 min per day)<br>LIPA (walking) (5 times 30 min per day)<br>MVPA (7 times 60 min per day)	45.9% of boys and 33.4% of girls<br>24.7% of boys and 20.2% of girls<br>58.1% of boys and 64.8% of girls<br>32.3% of boys and 20.1% of girls
Frömel et al., 2017 [26]	VPA (≥ 3 times 20 min per day)<br>MVPA (≥ 5 times 60 min per day)<br>Daily step count (11 000 steps per day)	41% of children<br>54% of children<br>24.2% of boys and 20.9% of girls
Frömel et al., 2016a [69]	500 steps per hour for school PA in PE lesson<br>MVPA during school PA (20 min per school time without PE lesson)<br>MVPA during school PA (20 min per school time with PE lesson)<br>MVPA (60 min per day)<br>Daily step count (11 000 steps per day)	83% of boys and 69% of girls<br>23% of boys and 18% of girls<br>74% of boys and 50% of girls<br>62.7% of boys and 59.9% of girls<br>54.1% of boys and 56.9% of girls
Frömel et al., 2007 [71]	VPA (≥ 3 times 20 min per day)	52.3% of boys and 40.4% of girls
Gába et al., 2017 [53]	MVPA (≥ 60 min per day)	22% of boys and 8% of girls
Groffik et al., 2020 [28]	500 steps per hour for school PA<br>at least 25% PA of overall school time<br>MVPA (20 min per school time)	39.8% of boys and 37.5% of girls<br>74.4% of boys and 62.1% of girls<br>30.9% of boys and 21.3% of girls
Hamřík et al., 2014 [80]	SB (≥ 4 hours per day)	65.7% of boys and 52% of girls
Jakubec et al., 2020 [58]	MVPA (≥ 60 min per day)<br>ST (< 2 hour per day)	18.3% of children and 6.8% of adolescents<br>11.0% of children and 26.2% of adolescents
Kalman et al., 2015a [59]	MVPA (7 times 60 min per day)<br>VPA (3 times 20 min per day)	From 24.8% to 29.5% among boys and from 14.3% to 23.3% among girls<br>50% of adolescents
Kalman et al., 2015b [30]	MVPA (≥ 60 min per day)	2002 – 31.1% of boys and 22.3% of girls<br>2006 – 26.6% of boys and 17.0% of girls<br>2010 – 27.4% of boys and 18.6% of girls
Kokko et al., 2018 [32]	MVPA (≥ 60 min per day)<br>VPA (≥ 4 times per week)	21% of adolescents<br>38% of adolescents
Kudláček et al., 2020 [46]	VPA (3 times 20 min per day)<br>MVPA (5 times 60 min per day)	67% of adolescents
Mitáš et al., 2020 [60]	Daily step count (11 000 steps per day)<br>MVPA (5 times 60 min per day)	2010–2017: From 67% to 39% among boys and from 60% to 42% among girls<br>2010–2017: 54% of boys and 47% of girls in
Mitáš et al., 2009 [70]	VPA (3 times 20 min per day)	54.6% of adolescents
Pavelka et al., 2012 [61]	MVPA (7 times 60 min per day)	46.7% of adolescents
Rubín et al., 2020 [62]	Combined movement guidelines (≥ 60 min per day of MVPA; < 2 hour per day of ST; 8–11 hour per day of sleep)	6.5% of children and 2.2% of adolescents
Sigmund et al., 2020a [76]	Daily step count (10 000 steps per day)<br>ST (≤ 2 hour per day)	47.3% of boys and 53.8% of girls<br>73.0% of boys and 74.2% of girls
Sigmund et al., 2018a [77]	Daily step count (11 000 steps per day for girls, 13 000 steps per day for boys)	59.5% of boys and 62.3% of girls

Sigmund et al., 2018b [63]	MVPA (≥ 60 min per day)	2002 – 32.2% of boys and 23.1% of girls<br>2006 – 27.6% of boys and 18.0% of girls<br>2010 – 28.1% of boys and 18.9% of girls<br>2014 – 25.6% of boys and 18.9% of girls
	ST (≤ 2 hour per day)	2002 – 23.2% of boys and 24.5% of girls<br>2006 – 11,7% of boys and 15.7% of girls<br>2010 – 24.4% of boys and 20.9% of girls<br>2014 – 13.3% of boys and 19.5% of girls
Sigmund et al., 2015a [64]	MVPA (≥ 60 min per day)	2002 – 32.2% of boys and 23.2%<br>2006 – 27.4% of boys and 17.9% of girls<br>2010 – 28.3% of boys and 19.2% of girls<br>2014 – 25.6% of boys and 19.2% of girls
	VPA (≥ 4 times 30 min per day)	2002 – n/a<br>2006 – 45.7% of boys and 24.4% of girls<br>2010 – 44.9% of boys and 30.3% of girls<br>2014 – 42.0% of boys and 33.6% of girls
	ST (> 2 hour per day)	2002 – 76.7% of boys and 63.2% of girls<br>2004 – 87.3% of boys and 76.6% of girls<br>2006 – 88.5% of boys and 83.1% of girls<br>2010 – 90.3% of boys and 80.2% of girls
Sigmund et al., 2014 [65]	MVPA (≥ 60 min per day) with PE lesson	47.3% vs. 30.9% normal-weight boys and 47.6% vs. 14.3% of overweight/obese girls
Sigmund et al., 2007 [43]	Daily step count (11 000 steps per day)	73% of children
Sigmundová et al., 2020b [102]	Daily step count (≥ 13 000/≥ 11 000 steps per day for 4–12-year-old boys/girls)	41.1% of boys and 46.9% of girls
	Daily step count (≥ 10 000 steps per day for 12–16-year-old adolescents)	52.8% of boys and 54.8% of girls
Sigmundová et al., 2018 [72]	Daily step count (13 000/11 000 steps per day for boys/girls)	67.7% of children
	ST (≥ 2 hour per day)	54.7% of children
Sigmundová et al., 2017 [84]	ST (≤ 2 hour per day)	12% of boys 23% of girls
Sigmundová et al., 2014 [73]	Daily step count (13 000/11 000 steps per day for boys/girls)	49.8% of boys and 55.6% of girls on weekdays and 39.2% of boys and 44.4% of girls on weekends
	ST (≥ 2 hour per day)	45.3% of adolescents on weekdays and 35.4% of adolescents on weekends
Sigmundová et al., 2013 [36]	MVPA (≥ 60 min per day)	2002 – 2010: From 30.8% to 25.7% among 11 years children<br>2002 – 2010: From 27% to 24.3% among 13 years children<br>2002 – 2010: From 22% to 19.6% among 15 years children
Sigmundová et al., 2011 [33]	Daily step count (11 000/9 000 steps per day for boys and girls)	54.5% of boys and 74% of girls
Šimůnek et al., 2017 [66]	LPA (5 times 30 min per day)	64% of secondary school students and 57% of university students
	MVPA (7 times 60 min per day)	29% of secondary school students and 28% of university students
Valach et al., 2017 [56]	VPA (3 times 20 min per day)	51.8% of boys and 37.7% of girls
Vašíčková et al., 2013 [90]	Daily step count (11 000 steps per day)	65.9% of boys and 64.7% of girls on schooldays and 42.6% of boys and 43.8% of girls on weekends
Vorlíček et al., 2019 [67]	MVPA (≥ 60 min per day)	59.9% of adolescents
Vorlíček et al., 2017 [74]	Daily step count (13 000/11 000 steps per day for boys and girls)	67.3% of adolescents
Whiting et al., 2020 [97]	ST (< 2 hour per day)	64.2% of children

Note. PA = Physical activity; SB = Sedentary behaviour; LPA = Light intensity physical activity; VPA = Vigorous physical activity; MPA = Moderate physical activity; MVPA = Moderate-to-vigorous physical activity; ST = Screen time; PE = Physical education.
